# Electroconvulsive Therapy-Induced Paroxysmal Atrial Fibrillation in Healthy Young Male

**DOI:** 10.7759/cureus.31989

**Published:** 2022-11-28

**Authors:** Brady Olsen, Julian S Trent, Brannon L Inman

**Affiliations:** 1 Emergency Medicine, San Antonio Military Medical Center, San Antonio, USA

**Keywords:** paroxysmal atrial fibrillation, ect anesthesia, cardioversion, atrial fibrillation (af), electroconvulsive therapy

## Abstract

Electroconvulsive therapy (ECT) is a widely used and highly effective treatment for psychiatric disorders. This is an overall safe option for the management of antidepressant-resistant depression; however, there are known possibilities of cardiac complications. The majority of documented cardiac-related complications due to ECT are found in patients who are middle-aged or older and generally have comorbidities, including prior myocardial infarction, known arrhythmias, hypertension, obesity, diabetes mellitus, family history of cardiac disease, alcohol abuse, and smoking. We present a case of an overall healthy, 21-year-old male with no prior cardiac disease who developed paroxysmal atrial fibrillation (AF) after a routine ECT treatment, his evaluation in the emergency department, treatment, and follow-up.

## Introduction

Atrial fibrillation (AF) is a cardiac arrhythmia in which poorly organized atrial depolarization of the heart does not conduct properly to the ventricles [1]. The key finding on electrocardiogram (ECG) is the lack of P waves leading to an irregularly irregular ventricular rhythm. This can be accompanied by an accelerated ventricular rate, leading to AF with rapid ventricular rate (RVR) [2]. There are many risk factors that may predispose a patient to AF, including increasing age, hypertension, diabetes, prior myocardial infarction, heart failure, obesity, smoking, alcohol abuse, family history of cardiac disease, among others [1]. AF is a clinically significant diagnosis that causes life-altering changes, as treatments may include the need for lifelong anti-arrhythmic medications, anticoagulation medications, cardiac ablation procedures, or pacemaker placement to control the rate and rhythm of the heart [3].

Electroconvulsive therapy (ECT) is a widely used treatment, and because it involves the use of medically induced generalized seizure activity, there are well-documented cardiac complications [4]. Patients with preexisting cardiac pathology are not barred from ECT, but extra consideration is needed prior to therapy in these types of patients [5]. ECT studies have shown cardiac effects such as sinus tachycardia, bradycardia, hypotension, and various ECG changes, including asystole or sinus arrest [6]. This is believed to be due to a sympathetic response as a result of ECT. This sympathetic response will cause a catecholamine release, leading to increased blood pressure, heart rate, and overall oxygen demand due to increased cellular metabolism. These hemodynamic changes cause stress on the heart, leading to the stretching of the atrial fibers, resulting in the development of AF [7]. AF has been described in a few case reports related to ECT, but has always been associated with patients of advanced age with several cardiac comorbidities. This case report will discuss a 21-year-old male patient with no prior cardiac history.

## Case presentation

The patient was a 21-year-old male residing in a long-term behavioral health facility for his diagnoses of post-traumatic stress disorder (PTSD) and treatment-resistant major depressive disorder. He was on several medications for his behavioral health issues, including aripiprazole, lamotrigine, hydroxyzine, sertraline, and zolpidem. He had a BMI of 33 but otherwise no past medical or surgical history. He denied any known allergies and stated that he believed someone in his family had AF, but denied personal cardiovascular problems.

**Figure 1:**
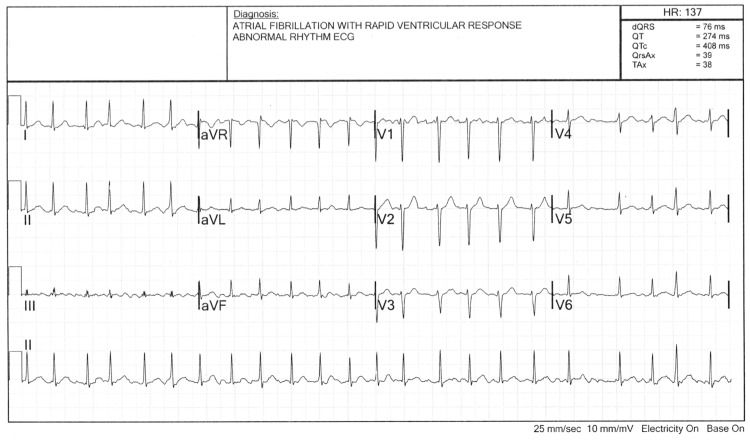
Electrocardiogram showing atrial fibrillation

He had received ECT several times in the past for his depression, and on the day of presentation underwent ECT at approximately 0700. He was pre-treated with 90mg brevital, given 80mg succinylcholine, and 2mg of midazolam. Immediately after recovering from sedation and treatment, he noted having palpitations, lightheadedness, and chest discomfort rated as 4/10. We did not have access to his post-procedural vital signs; however, he was noted to be in a normal sinus rhythm before being discharged back to his care facility. At approximately noon that same day, the patient was found to have an irregular heart rhythm by his care facility, who then transferred him via ambulance to the local emergency department. Upon arrival, he complained of the same symptoms as before. Upon initial ECG, he was in AF with RVR with a ventricular rate of 137, as seen in Figure 1. Laboratory analysis, including complete blood count, comprehensive metabolic panel, troponin, and a thyroid stimulating hormone level, were unrevealing. He was given 15 mg of diltiazem for rate control, after which his heart rate was reduced to 90 beats per minute, but he remained in AF. As the time of onset was known to be at 0700 during his ECT at the earliest, the decision was made to cardiovert him back to sinus rhythm. After the patient was consented, electrical pads were placed in the anterior/posterior placement, and he was given 10mg of etomidate via intravenous push. The first cardioversion was 120 joules, which had no effect. The second cardioversion at 200 joules resulted in conversion to normal sinus rhythm. Repeat EKG confirmed normal sinus rhythm with a rate of 67 bpm. The patient recovered well in the emergency department and was transferred back to his care facility.

Two weeks later, the patient followed up with cardiology. He received a transthoracic echocardiogram, which confirmed normal heart structure and function. A repeat EKG at that time showed that he was still in normal sinus rhythm. The patient denied any symptoms similar to when he was in AF.

## Discussion

This article presents a young, comparatively healthy patient with no prior cardiac abnormalities who was diagnosed with AF with RVR after ECT. His AF was persistent over several hours, and after being medically rate controlled finally required electrical cardioversion for rhythm control. Upon further cardiac workup, there was no structural abnormality or concerning underlying ECG findings to indicate a reason why he developed AF. 

Multiple case reports have been written about patients with new onset paroxysmal AF after ECT [5,7,8]. All of these patients either had significant cardiac conditions (including prior AF) prior to ECT, were of advanced age, or were spontaneously rate and rhythm controlled immediately after ECT with diltiazem administration. This case is significant for several reasons; the patient was very young, in a generally good state of health, and had significant symptoms while in AF. Other case reports deny the patients having any discomfort or chest pain and generally did not require cardioversion to attain rhythm control as our patient did.

It is important to know the long-term effects of ECT. Our patient was able to be successfully cardioverted back into normal sinus rhythm, but his future likelihood of AF is unknown. Several case reports have shown support of continued ECT after the development of AF [5,7,9]. Possible prevention strategies include beta-blockers administration prior to ECT. Labetalol and esmolol have both been used, with labetalol showing a stronger ability to protect against increases in blood pressure and heart rate during ECT [10].

## Conclusions

Although AF is a rare complication after ECT, this case report highlights the need for careful selection of patients prior to initiating ECT. Even young patients with seemingly healthy hearts need to be properly evaluated prior to treatment and monitored closely both during and after ECT, as symptoms may not be apparent until many hours after completion of ECT. Resuming ECT after the onset of paroxysmal AF, although not absolutely contraindicated, may be appropriate. These patients need to weigh the benefits of ECT with the associated cardiac risks. As discussed, there are medications that can be given prior to ECT to help reduce the risk of inducing AF, which may be beneficial in helping continue ECT when it is effective in reducing symptoms of depression.
